# Exophthalmos in a young woman with no graves’ disease – a case report of IgG4-related orbitopathy

**DOI:** 10.1186/s12886-018-0672-y

**Published:** 2018-01-12

**Authors:** Annamaria Erdei, Zita Steiber, Csaba Molnar, Ervin Berenyi, Endre V. Nagy

**Affiliations:** 1 Division of Endocrinology, Department of Medicine, Faculty of Medicine, University of Debrecen, Nagyerdei krt 98, Debrecen, 4032 Hungary; 2 Department of Ophthalmology, Faculty of Medicine, University of Debrecen, Nagyerdei krt 98, Debrecen, 4032 Hungary; 3 Institute of Pathology, Faculty of Medicine, University of Debrecen, Nagyerdei krt 98, Debrecen, 4032 Hungary; 4 Department of Radiology, Faculty of Medicine, University of Debrecen, Nagyerdei krt 98, Debrecen, 4032 Hungary

**Keywords:** IgG4-related orbitopathy, IgG4-related disease, Graves’ orbitopathy, Graves’ disease, Case report

## Abstract

**Background:**

Immunoglobulin G4-related disease (IgG4-rd) is characterized by lymphoplasmacytic infiltration and tissue fibrosis. Orbital manifestations of IgG4-rd may include unilateral or bilateral proptosis, cicatricial extraocular muscle myopathy, orbital inflammation and pain which may mimic ophthalmic Graves’ disease.

**Case presentation:**

A 25-year-old woman has been referred to the endocrinology clinic, 4 months after delivery, with suspected Graves’ orbitopathy. She has had bronchial asthma and recurrent skin rashes of unknown aetiology for the last 10 years and was treated for dacryoadenitis with steroid containing eye drops 5 years ago. During pregnancy she developed eyelid swelling. After delivery, eyelid redness and retrobulbar pain evolved. Proptosis was demonstrated by Hertel’s exophthalmometry. Orbital magnetic resonance imaging showed enlarged lateral and superior rectus muscles in both orbits. Thyroid function tests were in the normal range and no thyroid stimulating hormone (TSH) receptor autoantibodies were present. The eye muscle involvement pattern raised suspicion, and the high IgG4 level with positive histology of the lacrimal gland confirmed the diagnosis of immunoglobulin G4-related orbitopathy. Rapid improvement was observed following oral methylprednisolone.

**Conclusions:**

IgG4-related orbitopathy may mimic Graves’ orbitopathy. Euthyroid patients with no TSH receptor autoantibodies should be evaluated for immunoglobulin G4-related orbitopathy. Once IgG4-related orbitopathy is proven, other manifestations of IgG4-related disease have to be searched for; lifelong follow-up is warranted.

## Background

The most common orbital disease usually with exophthalmos is Graves’ orbitopathy, which is the extrathyroidal complication of Graves’ thyroid disease [1]. The annual incidence rate of Graves’ orbitopathy has been estimated at 16 cases per 100,000 women and 2.9 cases per 100,000 men in one rural Minnesota community [2]. Graves’ orbitopathy usually appears simultaneously with or soon after the development of thyrotoxicosis; however, rarely it may precede hyperthyroidism. The most common clinical features of Graves’ orbitopathy are upper eyelid retraction, oedema, and erythema of the periorbital tissues and conjunctivae, proptosis, dry ocular sensation, photophobia, double vision, and pressure sensation behind the eyes.

Beside detailed ophthalmological examination (best-corrected visual acuity, color vision, pupillary examination, ocular motility, Hertel’s exophthalmometry, intraocular pressure, adnexal examination, slit-lamp examination, dilated fundus examination) laboratory parameters that are necessary to confirm the diagnosis include: measurement of serum thyroid stimulating hormone, free thyroxin, and TSH receptor antibody levels. In euthyreoid Graves’ orbitopathy, TSH receptor antibody level is elevated without thyroid function abnormality. The diagnosis of Graves’ orbitopathy in most patients is obvious; however, exophthalmos can also be present in patients with lymphoproliferative disorders of the orbits, idiopathic orbital inflammatory syndrome, orbital myositis, severe obesity, Cushing’s syndrome, histiocytosis, granulomatosis with polyangitis, and IgG4-related orbitopathy [1, 3, 4]. Orbital magnetic resonance imaging, orbital computed tomography and/or single photon emission computed tomography can help to distinguish between the underlying causes [5–8].

## Case presentation

**Fig. 1:**
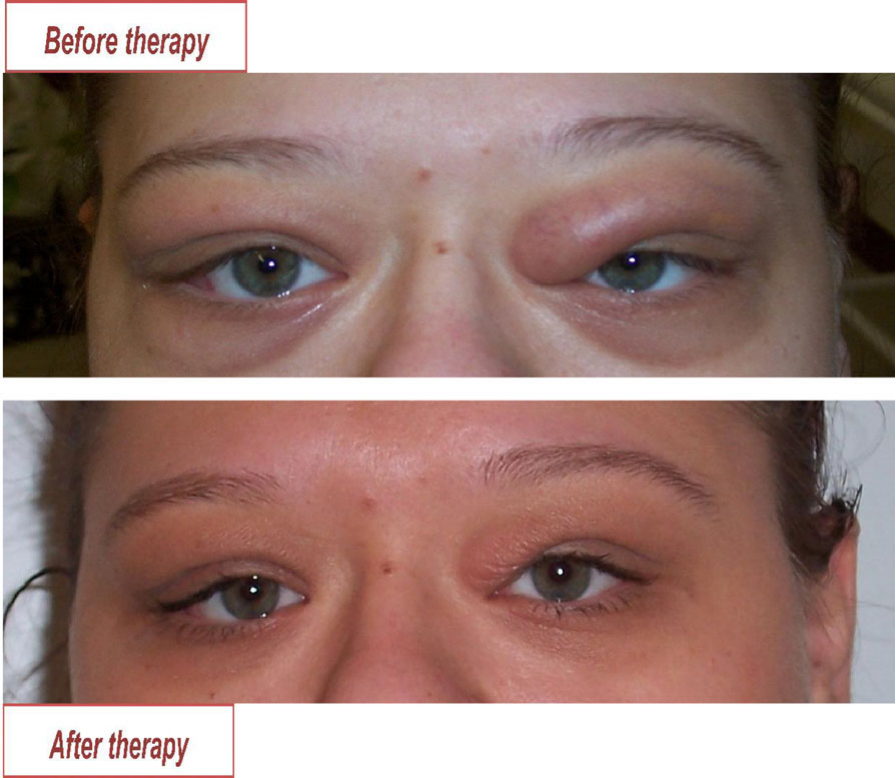
Changes in the condition of the eyes after 6 days oral glucocorticoid treatment

A 25-year-old woman presented in the endocrinology clinic, in October 2013, with eyelid swelling, redness and retrobulbar pain with suspected Graves’ orbitopathy after ophthalmological examination, 4 months after delivery (Fig. 1). Symptoms started during the third trimester of her pregnancy, in April 2013, which were considered pregnancy related phenomena and thus remained untreated. The patient did not have diplopia at the first visit, however, she complained about intermittent double vision starting 1 month later. In addition, she complained of a palpable “nodule” in the left side of her face.

**Fig. 2:**
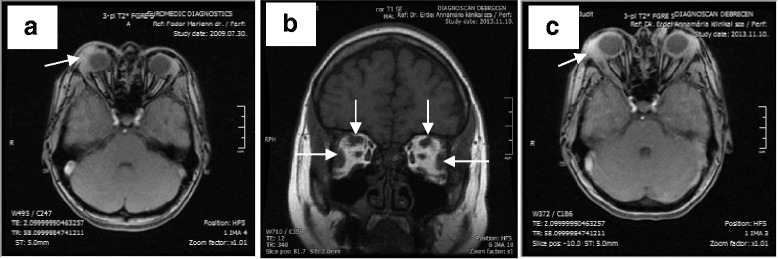
MRI of the orbits. **a**: T2 axial scan (in 2009). Inflammation in the right lacrimal gland and in surrounding connective tissue (arrow). **b**: T1 coronal scan (in 2013). The lateral and superior rectus muscles are enlarged in both orbits (arrows). **c**: T2 axial scan (in 2013). The arrow points to swollen lacrimal gland in the right orbit

The patient has had bronchial asthma and recurrent skin rashes of unknown aetiology for the past 10 years. Repeated examinations failed to find any evidence of an autoimmune disorder. Total IgG level was normal, while IgA and IgM were lower in 2008; with follow-up being recommended (Table 1). Dacryoadenitis was diagnosed by ophthalmological examination and MRI in 2009 (Fig. 2a). Her symptoms disappeared within a few days after using a steroid containing eye drops.

**Table 1:** Relevant laboratory values. Glucocorticoids were administered from January to March and from April to June, 2014

Laboratory parameters (with normal ranges)	May 2008	October 2013	January 2014	May 2014	July 2014	July 2015	June 2016	April 2017
TSH (0.3–4.2 mU/L)	1.25	3.36	1.72	2.02	2.1	2.17	2.62	2.23
free T4 (12–22 pmol/L)	13.7	14.6	14.8	13.4	15	15.2	13.4	14.2
free T3 (2.4–6.3 pmol/L)	4.4	5.1	4.9	4.9	4.1	5.2	5.1	4.6
TSH receptor antibody (<1 U/L, 1–2: grey zone)	–	1.0	<1	<1	–	<1	<1	<1
thyroid peroxidase antibody (<60 IU/ml)	<30	<30	<30	<30	–	<30	–	–
IgG4 (0.08–1.4 g/L)	–	–	9.4	3.03	1.29	10.5	14.9	12.6.
IgG (6.9–14 g/L)	12.31	–	9.9	4.87	5.5	10.9	12.9	12.8
IgM (0.4–2.4 g/L)	0.25	–	0.3	0.74	0.58	0.4	0.43	0.46
IgA (0.7–3.7 g/L)	0.61	–	0.5	0.63	0.6	0.6	0.65	0.5

On arrival at the endocrine unit in 2013, no signs or symptoms of hyperthyroidism were present. The thyroid gland was of normal size and without nodules. The palpable nodule on her face was identified, by ultrasound, as an unusual swollen limb of the left parotid gland, while thyroid ultrasound revealed no abnormalities. Physical examination revealed rashes on her legs. Proptosis values (measured by Hertel’s exophthalmometer) were 24 mm on the right side, and 21 mm on the left side. Visual acuity was 1.0 in both eyes, intraocular pressures were 16 mmHg and 20 mmHg on the right and left sides, respectively. Pupils were isocoric, equal and had normal reaction. Digital retropulsion did not suggest a mass or tumor in the retrobulbar space. Upon examination both the lower and upper eyelids were found to be markedly swollen and a swollen lacrimal gland became visible after the right upper eyelid had been lifted.

**Fig. 3:**
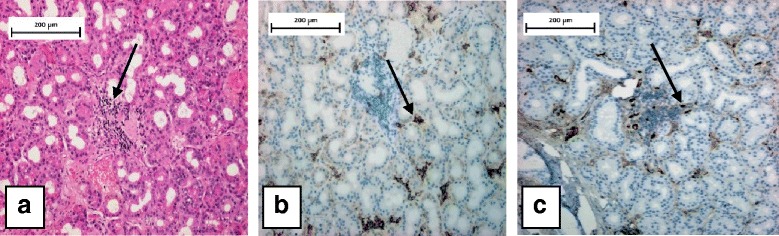
Histology of the lacrimal gland. **a**: Haematoxylin and Eosin staining. There is lymphoplasmacytic infiltration dominantly in the middle of the picture (arrow). **b**: CD138 immunostaining. Plasma cells are brown (arrow). **c**: IgG4 immunostaining. IgG4-positive plasma cells brown (arrow)

Orbital MRI showed enlarged lateral and superior rectus muscles, and eyelid oedema and swollen lacrimal gland in both orbits while the typical MRI features of Graves’ orbitopathy (proptosis, enlarged extraocular muscles, most frequently the inferior and medial rectuses, excessive amount of orbital connective tissue) were missing (Fig. 2b, c). Serum levels of TSH and thyroid hormones were in the normal range. TSH receptor antibody and thyroid peroxidase antibody levels were not elevated (Table 1). Based on her medical history, laboratory and imaging results, the possibility of IgG4-rd was suspected. A markedly elevated IgG4 level was observed (Table 1). Histological examination of the right lacrimal gland confirmed the diagnosis of Ig4-related orbitopathy, with both CD138 (the unique cell surface marker of plasma cells) and IgG4 immunohistochemical stainings showing plasmacytic infiltration in the same localisation (Fig. 3), and excluding other IgG4 positive conditions thereby confirming the diagnosis of IgG4-related orbitopathy.

Placed on oral corticosteroid administration (methylprednisolone, 16 mg daily) a rapid improvement in both the eye symptoms and skin rashes was observed within a few days (Fig. 1). However, after gradual tapering of the corticosteroid therapy (from January to March 2014), her ocular symptoms relapsed. After a second course of oral methylprednisolone for 8 weeks (from April to June 2014), the parotid gland enlargement disappeared and she remained asymptomatic for the next 24 months. However, IgG4 level started to rise during follow-up and in April 2017, periodic skin rashes appeared on the legs. As IgG4 level rose, skin biopsy was suggested, which she did not consent to. Her skin symptoms are currently under control with glucocorticoid-containing ointments. Should systemic treatment for the IgG4-rd be necessary in the future, rituximab therapy could be considered, since she has already rejected any future steroid courses fearing weight gain as one of its side effects [9]. We assume that the swollen parotid gland and skin symptoms are additional manifestations of IgG4-rd, however, without biopsy this remains unproven.

## Conclusions

IgG4-rd is a recently described and increasingly recognized entity [10] which may present with involvement of the eyes, termed IgG4-related orbitopathy [4, 11]. IgG4-rd usually affects more than one organ, typically the orbits, salivary glands, lungs, pancreas, biliary ducts and retroperitoneal tissue. Orbital involvement most frequently includes the lacrimal glands and extraocular muscles, however, infraorbital and supraorbital nerve enlargement may also be detected [12]. In a series of 1014 orbital lymphoproliferative disease cases, the incidence of IgG4-related orbitopathy was found to be 21.6% [13]. Features of IgG4-rd include tumor-like swelling of involved organs, lymphoplasmacytic tissue infiltration enriched in IgG4-positive plasma cells, storiform fibrosis and obliterative phlebitis [4, 10, 11]. The number of IgG4-positive plasma cells per high-power field (HPF) that is regarded as consistent with or suggestive of IgG4-rd varies somewhat from tissue to tissue. Generally, the minimum for making the diagnosis for most tissues is from 30 to 50 IgG4-positive cells/HPF. However, in the lacrimal gland, 10 IgG4-positive plasma cells/HPF may be sufficient for the diagnosis [13–15]. Storiform fibrosis and obliterative phlebitis are more typical of the systemic pathology; however, they are not always present in the orbital disease. A specific autoantigenic target has not been identified; furthermore, it is not clear whether the IgG4 antibodies are pathogenic. In 2009, diagnostic criteria for IgG4-rd were proposed, and comprehensive diagnostic criteria are currently in use [15, 16]. In patients with typical clinical features and organ involvements both measurement of serum IgG4 levels and tissue biopsy are recommended [14–16]. Elevated serum concentrations of IgG4 are found in 60–70% of patients with IgG4-rd, but this finding is not specific, as it can also be associated with Churg-Strauss syndrome, sarcoidosis and allergic diseases [17].

All patients with symptomatic active IgG4-rd require treatment. Glucocorticoids are the first-line agents for remission induction. Following a successful course of induction therapy, certain patients benefit from maintenance therapy. Repeated glucocorticoid courses are indicated in patients who relapse following successful remission induction. Most patients respond to glucocorticoids within several weeks, typically with symptomatic improvement, reduction in the size of masses or enlarged organs, improvement in organ function, and a decrease in serum levels of IgG4 [4]. However, some require a few months to respond, while others relapse and others who respond less well or not at all initially. In those who are resistant to glucocorticoids, or have side effects or contraindications to glucocorticoid therapy, rituximab is another therapeutic option [9].

Neither the natural course nor the prognosis of IgG4-rd is well-understood. Spontaneous improvements can be seen, but the disease often relapses if left untreated. Symptoms respond dramatically to glucocorticoids, but relapses are common following discontinuation of therapy. Significant organ dysfunction may arise from uncontrolled and progressive inflammatory and fibrotic changes in affected tissues. Currently, it is uncertain if there is an increased risk of malignancy in patients with IgG4-rd, however, lymphomas occur more frequently in patients with IgG4-related disease [10, 16].

The prevalence of the disease could be more frequent than it is diagnosed [13]. There is enhanced attention towards IgG4-related orbitopathy in Japan [4, 13], as well as few published cases in Europe.

As far as clinical manifestations are concerned, cutaneous presentations, similar to the present case, may be the first sign of IgG4-rd [18].

The most typical manifestations of IgG4-related orbitopathy are the usually easily detectable dacryoadenitis, swollen eyelid and involvement of extraocular muscles. The external eye muscle involvement pattern on MRI is distinct from Graves’ orbitopathy, where the inferior and medial rectuses are the most frequently involved muscles [5]. In IgG4-related orbitopathy these muscles are often spared, as seen in our case (Fig. 2b) [12]. Less commonly, compressive optic neuropathy secondary to mass effect from inflammatory lesions may result in visual loss, afferent pupillary defect, or visual field defects. Mass effect can particularly occur in patients with infraorbital nerve enlargement, which is defined as the infraorbital nerve being greater in diameter than the optic nerve. Infraorbital nerve enlargement is significantly correlated with elevated serum IgG4 levels. Sensory loss is extremely rare. Other less common manifestations are frontal nerve and perioptic nerve lesions. Involvement of the nasolacrimal duct system can cause epiphora. In rare cases of IgG4-related orbitopathy causing scleritis, patients may present with conjunctival and scleral injection with blurred vision [18].

In our patient, skin symptoms and salivary gland enlargement have also been components of IgG4-rd. Although only the lacrimal gland was biopsied, improvement of the skin and salivary gland lesions after glucicorticoid therapy was indirect evidence of the involvement of these organs. Symptoms which were typical of bronchial asthma may have been caused by IgG4-rd. Possible pulmonary manifestations of this condition are nonspecific interstitial pneumonia, sclerosing mediastinitis, mediastinal or hilar adenopathy, massive pleural effusion, nodular pleural lesions and pulmonary arterial hypertension [19, 20]. During follow-up, we did not detect any of these abnormalities.

The case presented here is rather unique as the patient is a young woman, since IgG4-rd occurs predominantly in elderly men [11, 12]. In her case age would have been rather characteristic of Graves’ orbitopathy. Data on the association of IgG4-rd and Hashimoto’s or Riedel’s thyroiditis exist [21], but similar association with Graves’ disease has not been described. Notably, one group found elevated IgG4 levels in 6.4% of 109 patients with Graves’ disease. This subgroup of patients was significantly older, their symptoms were manageable with small doses of antithyroidal drugs and they were prone to be hypothyroid after treatment [22]. It has recently been shown that IgG4 levels may be elevated in newly diagnosed Graves’ disease patients compared with euthyroid subjects and in the presence of Graves’ orbitopathy compared with the absence of Graves’ orbitopathy [23]. However, the IgG4 rise in IgG4-rd is more marked. Corticosteroid treatment is the standard therapeutic option for both Graves’ orbitopathy and IgG4-related orbitopathy, but unlike in Graves’ orbitopathy, marked response to low corticosteroid doses is typical in IgG4-related orbitopathy [4].

IgG4-related orbitopathy may easily be mistaken for Graves’ orbitopathy [24]. All patients with suspected euthyroid Graves’ orbitopathy in whom TSH receptor autoantibodies are not present have to be evaluated for IgG4-related orbitopathy. Once IgG4-related orbitopathy is proven, other manifestations of IgG4-rd have to be searched for; lifelong follow up is required. Immunoglobulin G4-related disease is an increasingly recognized syndrome of unknown etiology. IgG4-rd could affect many organs and tumor-like swelling of the involved organs is common. Several of the manifestations typically occur in the same patient. The diagnosis of IgG4-rd is based upon biopsy findings (IgG4 positive lymphoplasmacytic infiltration and tissue fibrosis). Serum IgG4 level should be measured, and isolated elevated levels support the diagnosis, although it is not fully diagnostic without histological examination. Due to various manifestations of this new entity, a multidisciplinary approach is warranted.

## Abbreviations

HPF: High power field
IgG4: Immunoglobulin G4
IgG4-rd: Immunoglobulin G4-related disease
MRI: Magnetic resonance imaging
TSH: Thyroid stimulating hormone
